# Trends in the HIV related hospital admissions in the HAART era in Barbados, 2004–2006

**DOI:** 10.1186/1742-6405-4-4

**Published:** 2007-03-07

**Authors:** Alok Kumar, Krishna R Kilaru, Shelly Sandiford, Sheila Forde

**Affiliations:** 1 School of Clinical Medicine and Research, The University of the West Indies (Cave Hill), Barbados; 2 Martindales Road, Saint Michael, Barbados; 3 STI Program, Sir Winston Scott Polyclinic, Ministry of Health, Barbados; 4 The Queen Elizabeth Hospital, Barbados; 5 The Ladymeade Reference Unit, Ministry of Health, Barbados

## Abstract

**Background:**

To investigate the reasons for hospitalizations and its outcome in the era of HAART in Barbados. This report also describes the profile of the HIV infected persons who are hospitalized in the HAART era.

**Methods:**

This is a retrospective study of HIV related admissions in this country. We examined the admission case notes of all the adult admissions to the Queen Elizabeth Hospital where one of the discharge diagnosis was HIV infection during the April 2004 through March 2006. Data collected included patients' profile, including the date of diagnosis of HIV infection, outcome of the current admission in term of discharge or death and the final diagnosis at the time of discharge or death.

**Results:**

Over the 24 months period there were 431 adult admissions to the medical wards of the Queen Elizabeth Hospital where one of the discharge diagnosis was HIV infection and this accounted for 5.9% of all medical admissions. 258(60%) admissions were in persons who were known to be HIV infected prior to the current admission, where as diagnosis of HIV infection was made for the first time during the current admission in case of remaining 76(47.5%) cases. Nearly half of those hospitalized, had a CD 4 cell counts of < 200/μL. Over all, opportunistic infection was the commonest (35%) discharge diagnosis, followed by serious bacterial infections, anemia and HIV nephropathy. The outcome of these admissions was death in 30 (14.2%) cases where as patient was discharged out in the remaining 181 (85.8%) cases. Of the medical admissions with HIV as one of the discharge diagnosis during the period April 04 through March 05, 43% were newly diagnosed HIV infection and the corresponding figure for the period April 05 through March 06 was 35% (P = 0.54). During the April 05 through March 04 significantly higher proportion of HIV infected adults had Anemia with a Hemoglobin less than 10 g/dL (P = 0.044), HIV related nephropathy (P = 0.0003), HAART toxicity (P = < 0.0001) and a Non-AIDS related conditions (P = 0.043) as one of the final discharge diagnosis.

**Conclusion:**

A significant proportion of patients admitted with HIV infection were the newly diagnosed and severely immuno-supressed. An opportunistic infection continues to be the commonest discharge diagnosis, although there was a growing trend in the proportion of the discharge diagnosis being HAART toxicity and Non-AIDS related conditions. Over all hospitalization of HIV infected persons still carries a significant risk of mortality.

## Background

The introduction of HAART has led to a decline in the overall hospitalizations rates as well as a reduction in the morbidity from the HIV infection [1-3], however, this decline has been unevenly distributed and inconsistent [4-8]. There are reports that have noted a plateau effect or even an increased hospitalization due to relative increase in hospitalizations for non-HIV related reasons, such as drug toxicity, chronic liver disease, and non-HIV associated neoplasm [4,5]. It is clear that the interactions of morbidity, mortality, and hospitalizations due to HIV disease remain complex in the HAART era and may vary across various demographic and geographic groups. However, most reports of hospitalization from HIV infection in the HAART era are from the developed countries [1-5]. There are very few published studies on HIV related hospitalization from the developing countries [9-11]. All these reports from the developing countries are from settings where HAART was not used [9-11].

The impact of the reduction in HIV/AIDS related morbidity and hospitalization in the HAART era in the Caribbean populations has not been characterized to date and there is no published report from the English speaking Caribbean countries on this subject. Barbados is one of the English speaking Caribbean countries which has made great progress in tackling this HIV epidemics over the past decade especially in the prevention of mother to child transmission of HIV and in the treatment of HIV infected persons after the introduction of HAART in early 2000 [12,13]. We investigated all the hospital discharges in Barbados, where one of the discharge diagnoses was HIV/AIDS, to identify the causes of hospitalization among the HIV infected persons in the HAART era and to describe any emerging trend.

## Results

Over the 24 months period there were 431 adult (people older than 16 years) admissions to the medical wards of the Queen Elizabeth Hospital (QEH) where one of the discharge diagnosis was HIV infection. There were 352 adults who were admitted to the QEH on one or more occasions accounting for these 431 admissions where HIV/AIDS was at least one of the final diagnoses. There were 7319 adult who were admitted to the medical wards of the QEH during the same period. Admissions in HIV infected persons accounted for 5.9% of all medical admissions to the QEH. Table 1 show the characteristics of the HIV infected persons hospitalized during the study period. The majorities (92%) of patients were Afro-Caribbean, and their median age at the time of hospitalization was 41 years (Range 16 – 71 years). Of the 352 adults who were admitted during the study period and who had HIV infection as one of the discharge diagnosis, 58.8% were males, 14.2% were men who had sex with men (MSM), 12.6% smoked marijuana and/or cocaine, and none were intravenous drug users (IDU). During the 24 months study period 53 (15%) patients had multiple admission (Median number of admissions = 3, Range - 2 to 5 admissions) and accounted for 30% of all the admissions.

**Table 1:** Baseline characteristics for the 352 HIV infected adults who were admitted during the study period.

Age Group	
≤ 30	71 (20.0)
31–50	219(62.2)
> 50	62 (17.8)
Gender	
Females	145 (41.2)
Males	207 (58.8)
Self-Described Sexual orientation	
Heterosexual	201 (57.1)
Homosexual men	19 (5.4)
Bisexual men	31 (8.8)
Bisexual females	7 (2.0)
Not disclosed	93 (26.4)
Drug abuse	
None	178 (50.6)
Marijuana smoking	33 (9.4)
Cocaine smoking	9 (3.2)
Not disclosed	132 (37.5)

Of the 431 adults admissions to the QEH with the diagnosis of HIV/AIDS, in 258 (60%) admissions the person was known to be HIV infected prior to the current admission, where as diagnosis of HIV infection was made for the first time during the current hospitalization in case of remaining 173(40%) of the hospitalizations. Of those hospitalized and who had a CD4 cell counts done (324 patients) with in 2 weeks of the time of admission, 207(48%) had a CD 4 cell counts of less than 200/μL and 215(50%) of those who had a viral load estimated had a viral load value of over 50, 000 copies/ml (Table 2). For those 258 patients known to be HIV infected at the time of their current hospitalization, median duration of time since their diagnosis was 31 months (Inter Quartile Range, 9 – 61 months) and 153(59%) were on HAART for a median duration of 10 months (Inter Quartile Range, 3 – 24 months). In 31% of those on HAART, adherence was categorized as being poor (taking less than 90% of the prescribed medications). Both absence of prior diagnosis of HIV infection (newly diagnosed HIV infection) and absence of prior HAART was associated with significantly (P = 0.003) higher risk of having a CD4 cell counts < 200 at the time of hospitalization.

**Table 2:** Clinical and laboratory characteristics of the 431 HIV – related adult admissions to the QEH.

	April 04-March05 N = 282	April 05-March06 N = 149	Over all N = 431
CD4 cell count at admission			
≤ 200/μL	139 (49)	58 (46)	207 (48)
≥ 200/μL	68 (24)	49 (33)	117 (27)
Not available	75 (27)	32 (21)	107 (25)
Viral load at admission			
< 50 copies/ml	32 (11)	28 (19)	60 (14)
50 – 49999 copies/ml	28 (10)	20 (13)	48 (11)
50000 – 149999 copies/ml	76 (27)	29 (20)	105 (24)
≥ 150000 copies/ml	74 (27)	36 (24)	110 (26)
Not available	69 (25)	36 (24)	108 (25)
Prior HIV diagnosis			
Yes	161(57)	97 (65)	258 (60)
No	121 (43)	52 (35)	173 (40)

N = Total number of admissions.

Over all, an opportunistic infection was one of the discharge diagnoses in 35%, followed by serious bacterial infections (27%), anemia (15%) and HIV nephropathy (13%) in order of frequency (Table 3). In 98 cases, opportunistic infection was the primary diagnosis where as serious bacterial infection was the primary diagnosis in 40 cases of hospitalizations. Common opportunistic infection as primary diagnosis included cerebral toxoplasmosis, PCP, disseminated herpes infection and Cryptococcus meningitis. A non-HIV/AIDS-related condition was the primary final diagnosis in 88 admissions (22%). Median duration of the hospital stay was 9 days (Inter Quartile Range, 4 – 18 days).

**Table 3:** Final discharge diagnosis for the 431 HIV – related adult admissions to the QEH.

	April 04-March 05 N(%)	April 05-March 06 N(%)	Over all N(%)
Opportunistic infections	104 (37)	47 (31)	151 (35)
Serious bacterial infections	69 (24)	48 (32)	117 (27)
Anemia (Hemoglobin < 10 g/D)	39 (14)	25 (17)	64 (15)
Nephropathy	24 (8)	34 (23)	58 (13)
Other AIDS related illness	21 (7)	25 (17)	30 (7)
HAART toxicity	9 (3)	34 (23)	22 (5)
Non-AIDS related illness	52 (13)	42 (28)	88 (20)

Trend analysis of the CD4 cell counts and the viral loads at the time of admission for the HIV infected persons during April 04 through March 05 compared with those during the April 05 through March 06 were not significantly different (P = 0.45). Of the 282 adult medical admissions with HIV as one of the discharge diagnosis during the period April 04 through March 05, 43% were newly diagnosed HIV infection and of the 139 adult medical admissions with HIV as one of the discharge diagnosis during the period April 05 through March 06, 35% were newly diagnosed HIV infection (P = 0.54). During the April 05 through March 04 significantly higher proportion of HIV infected adults had Anemia with a Hemoglobin less than 10 g/dL (P = 0.044), HIV related nephropathy (P = 0.0003), HAART toxicity (P = < 0.0001) and a Non-AIDS related conditions (P = 0.043) as one of the final discharge diagnosis. Proportion HIV infected adults with an Opportunistic infection and serious bacterial infection (Pneumonia, Septicemia, Pyo-Meningitis, Pyelonephritis, Endocarditis or infections of bone and joints or deep seated tissues) as one of the final discharge diagnosis during the April 05 through March 06 were not significantly different from those during the April 04 through March 05.

The outcome of these admissions was death in 54 (12%) cases where as patient was discharged out in the remaining 377 (88%) cases. Death outcome was more common in persons with history of poor adherence to HAART (21%) and those with a CD4 cell counts of < 200/μL at the time of their admission (18%) compared to persons with good adherence (9%) and those who had a CD 4 cell counts of ≥ 200/μL at the time of their admission (6%), and these differences were statistically significant (P = 0.04 and 0.04 respectively). The death rate among the persons who were diagnosed to be HIV infected during the current admission (11%) and those not on HAART (14%) was higher as compared to those with prior HIV diagnosis (15%) and those who were on HAART at the time of their admission (11%). However, these differences were statistically not significant. Of the 377 discharges among the HIV infected persons during the study period, a follow up visit to the LRU with in 6 weeks of discharge was recorded in 292 (77%) cases.

## Discussion

We found that HIV-related hospitalizations in the only public hospital in Barbados constituted a significant proportion of all medical admissions in adults. Multiple hospitalizations were clustered in over one-eighth of the HIV infected persons. Majority of our admitted patients were heterosexual males in the age group 31 to 50 years (Table 1) which is consistent with the socio-demographic profile of the newly diagnosed HIV infected adults in this country [16]. Nearly half of all the HIV related admissions were in persons not known to be HIV infected prior to this admission. Also, nearly half of the adults admitted to the medical wards and who had HIV infection as one of the discharge diagnosis, had a CD4 cell count value < 200 and a viral load value of over 50,000 copies/ml at the time of their admission. These data are consistent with the fact that late diagnosis of HIV infection continues to be a major problem in the HAART era and that this may offset the benefits of HAART in reducing the morbidity and mortality form HAART in this population. An earlier study form this population when HAART was being introduced in this country had also reported the late diagnosis as a significant problem [16]. Late diagnosis of HIV infection is common despite the universal availability of voluntary testing facility for HIV and anti-retroviral therapy to all Barbadian public free of cost at the point of delivery. Finding from the studies such as this one highlights the occurrence of late diagnosis of HIV in this country and should be used by health educators and counselors to encourage the general public for frequent and periodic testing for HIV on a voluntary basis. Indirectly and perhaps more importantly, these findings highlight prevalence of high degree of stigma and discrimination prevalent in the society [17]. The issue of stigma and discrimination of the HIV infected persons is further compounded by the mode of delivery of the health care and treatment for these people in this country. The centralized HIV/AIDS center which was meant to be the "one stop shop" for all the health care need of the HIV infected persons in this country may proving to be a double-edged sword, where by people may not be seen in a place popularly associated with the HIV care, in a small society such as this where practically everybody knows everybody else.

Consistent with the findings of a high proportion of the hospitalization occurring in newly diagnosed HIV infected individuals who were not on any HAART and who had a low CD4 cell count, opportunistic infection remains the single most frequent cause of HIV related hospitalization in this country in the era of HAART (Table 3). Low CD4 counts, AIDS, and no current use of highly active antiretroviral therapy (HAART) are strongly correlated with hospitalizations especially those due to opportunistic infections [4,7]. What we also found is that a significant proportion of the adults with the prior diagnosis of HIV infection and who were hospitalized had severe immuno-supression with a CD4 cell counts < 200/μL (36%) and over two-thirds of these patients were on HAART at the time of hospitalization. Poor adherence and emergence of resistance to the HAART regimen may be the possible reason for the immununologic failure in these patients on HAART. Nearly one-third of the patients on HAART were described to have poor adherence to the HAART regimen. Of those who had poor adherence, over four-fifths were on their third HAART regimen and all except one were on their second HAART regimen. After failing the first regimen which consisted of a combination of Combivir and Nevirapine, Nevirapine was empirically replaced by a protease inhibitor. No resistance testing was done in any of these patients. There are well controlled prospective study to show HIV related admissions from opportunistic infections occurred significantly more often in patients ignorant of their HIV status, those who did not have follow up and those that were non-compliant with their HAART [18]. However, slow immune recovery could account for immuno-suppression in many patients [19].

Along with the existing problems of late diagnosis of the HIV infections with advance stage of the HIV infection and non-adherence to therapy among those known to be HIV infected and on HAART with possible resistance and treatment failure contributing to many of these admissions, there is a growing trend toward increasing admissions for HAART toxicity and Non-AIDS related conditions. Also, as persons with AIDS on HAART are living longer, there is an increase in the nutritional problems such as anemia, and chronic diseases such as nephropathy.

There are some limitations to this retrospective observational study. Missing data were common, despite efforts to complete the data set. In particular, one third of patients were lacking viral load and CD4 T cell count data, and complete treatment data for the entire cohort are lacking. However, although, this is a hospital based study, this cohort of HIV infected persons requiring hospitalization is representative of the entire population of Barbados as QEH is the only hospital that provides inpatient care to the HIV infected persons in this country. This could be seen as strength of this study.

In conclusion, a significant proportion of patients admitted with HIV infection were the newly diagnosed and were more likely to be severely immuno-supressed with an increased risk for mortality. An opportunistic infection continues to be the commonest discharge diagnosis, although there was a growing trend in the proportion of the discharge diagnosis being HAART toxicity and Non-AIDS related conditions. Over all hospitalization of HIV infected persons still carries a significant risk of mortality. The clustering of hospitalizations in a small number of patients may enable the development of support programs targeted towards these "hospitalization-prone" patients to reduce recidivism.

## Methods

Barbados is one of the smaller countries in the English speaking Caribbean, with an estimated 2001 population of 266,800 and a 2001 estimated per capita gross national product of US$ 14010. Crude mortality rate for Barbados for 2001 was at 8.3 per 1000 population [14]. The adult prevalence rate of HIV in this country is at 1.75%, with the male: female ratio of 2:1 [15]. The Government of Barbados views health care as a fundamental right of all Barbadians and aims to provide comprehensive health care to all its citizens, through its elaborate government controlled health care facilities, free of cost at the point of delivery. There is a provision for voluntary counseling and testing for HIV and an excellent facility for the follow up care and treatment of all HIV infected individuals in this country including provision for regular CD4 cell counts and Viral load estimation to follow the course of this illness. HAART is available for all eligible HIV infected persons since 2001. All these facilities are provided through a centralized HIV follow up clinic at the LRU, free of cost.

The Queen Elizabeth Hospital (QEH) is the only hospital with facility for the in-patient care for HIV infected persons in the whole of Barbados. Hospital maintains a detailed record of all inpatients admissions. Ambulatory care and management of HIV infected persons in this country is coordinated and provided through a centralized HIV clinic called the Ladymeade Reference Unit (LRU). Prior to 2002, this centralized HIV clinic used to operate from the Respiratory Unit (RU) at the QEH. Care and treatment including HAART has been available for the HIV infected persons in Barbados since 2002 at no direct cost at the point of delivery. There were a total of 850 HIV infected adults registered at the LRU from among an estimated 2650 HIV infected adults living in Barbados. Unique patient identification unit numbers were used to identify the cohort across both services and databases.

This is a retrospective study. All the admissions to the medical wards of the QEH during the April 2004 through March 2006 where one of the discharge diagnoses was HIV/AIDS were included in this study. Although one cannot be sure that all of the other patients were not HIV infected, it is a routine practice at the Queen Elizabeth Hospital to Screen all adult inpatients on the medical wards for the HIV after counseling and where the patient consents for the test. Admission charts for all these admissions were reviewed by one of the authors to extract the relevant data. Data collected included patient related information, history of drug abuse, self-described sexual orientation, date of diagnosis of HIV infection, CD4 counts and Viral load values at the time of diagnosis and at the time of the current admission and whether they received HAART prior to the current admission. For persons who were on HAART at the time of current admission, duration of HAART, nature of HAART regimen, failure of any previous regimen and adherence to HAART regimen was recorded. Outcome of the current admission in term of discharge or death and the final diagnosis at the time of discharge or death was noted.

Outcome variables were the frequency and causes of hospitalization among HIV infected persons, duration of hospital stay, out come of hospitalization in terms of death or discharge. Proportion of HIV related hospitalizations in persons not known to be HIV infected prior to their admission and the proportion oh HIV related admissions occurring in persons attending the LRU for their follow up care was also measured. Predictor variable includes- age, gender, CD4 cell counts and viral load values at the time of diagnosis, CD4 cell counts and viral loads at the time of current hospitalization, and history of being on HAART. Bi-variate relationships between variables were investigated using the chi-square test of association for nominal variables. A 0.05 significance level was used for all statistical tests. Data was stored in a specially designed Microsoft Access database and was analyzed using SPSS statistical soft ware package for windows version 11. Microsoft excel was used for the generation of all graphs and tables.

## List of abbreviations

HIV – Human Immunodeficiencey Virus

AIDS – Acquired Immunodeficiency Syndrome

HAART – Highly Active Anti-Retroviral Therapy

QEH – Queen Elizabeth Hospital

LRU – Ladymeade Reference Unit

## Authors' contributions

AK designed the study, carried out analysis and writing the manuscript.

KRK helped with the design of the study, entered the date into the computer database and cross checked the manuscript.

SS helped with the collection of data.

SF helped with the collection of data.
